# The Ta Seed-Buffer Layer Microstructure and Its Influence on the Magnetic and Structural Parameters of CoFeB/MgO Layers

**DOI:** 10.3390/ma18245558

**Published:** 2025-12-11

**Authors:** Jarosław Kanak, Monika Cecot, Witold Skowroński, Antoni Żywczak, Marta Gajewska, Jerzy Wrona, Wiesław Powroźnik, Maciej Czapkiewicz

**Affiliations:** 1 Institute of Electronics, AGH University of Krakow, Al. Mickiewicza 30, 30-059 Cracow, Poland; kanak@agh.edu.pl (J.K.); monika.cecot@agh.edu.pl (M.C.); skowron@agh.edu.pl (W.S.); wpowroz@agh.edu.pl (W.P.); 2 Academic Centre for Materials and Nanotechnology, AGH University of Krakow, Al. Mickiewicza 30, 30-059 Cracow, Poland; zywczak@agh.edu.pl (A.Ż.);; 3 Department of Mechanical Engineering, Faculty of Engineering, Otemon Gakuin University, Osaka 567-8502, Japan; 4 Singulus Technologies AG, Hanauer Landstrasse 103, 63796 Kahl am Main, Germany; jerzy.wrona@singulus.de

**Keywords:** X-ray diffraction, crystallization, texture, dead layer, buffer layers, magnetic tunnel junction

## Abstract

In this paper, we discuss the structural and magnetic properties of Ta(*d*)/Co_40_Fe_40_B_20_/MgO/Ta multilayers. The CoFeB wedge layer was deposited on three buffers differing in Ta layer thickness: *d* = 5, 10, and 15 nm. A structural analysis showed that the Ta seed-buffer of 5 nm was amorphous, whereas thicker Ta grew in a β-tetragonal disordered structure. X-ray reflectivity measurements revealed that the Ta/CoFeB interface roughness for annealed samples ranged from 0.55 to 0.67 nm for a sample with a 0.85 nm CoFeB layer and decreased to approximately 0.47 nm for thicker CoFeB layers, while the average interface CoFeB/MgO thickness was about 0.2–0.3 nm. The morphological roughness of the amorphous single 5 nm Ta layer was the lowest, whereas crystalline grains in thicker Ta buffers induced higher roughness. The 5 nm thick MgO layer exhibited a strong (001)-oriented texture, which was the highest for the smoothest 5 nm Ta buffer. The magnetic dead layer thickness for the annealed sample with a 15 nm Ta buffer was 0.39 nm and increased with the decrease in the Ta buffer thickness. Temperature-dependent measurements offered further insight into the diffusion processes and the formation of the magnetic dead layer (MDL) at the Ta/CoFeB interface.

## 1. Introduction

Magnetic tunnel junctions with ultrathin CoFeB electrodes can exhibit perpendicular magnetic anisotropy (PMA) and high tunnel magnetoresistance (TMR) [1]. These properties, which grant an optimal energy barrier, a high signal-to-noise ratio, and a low switching current, make them promising candidates for high-storage-density magnetic random access memory (MRAM) cells due to their non-volatility and fast writing speed [1,2,3]. It has been previously shown that buffer layers strongly affect the magnetic and structural properties of magnetic multilayers [4,5,6,7], which can be crucial for obtaining the desired parameters, such as crystallization direction, crystalline texture, and mixing at the interface or magnetic dead layer (MDL).

In spintronic structures, e.g., magnetic tunnel junctions (MTJs), the magnetic dead layer reduces the effective thickness of the ferromagnetic layer and consequently weakens the total magnetic moment. It can also affect the magnetic anisotropy, the TMR ratio, and even the thermal stability of MRAM memory elements.

The thickness of MDL depends on the material and crystallographic structure of the buffer layers [8,9]. In our case, the interfacial PMA arises from both intermixing at the Ta/CoFeB interface [6,10,11] and from hybridization of transition metal 3d and oxygen 2p orbitals [12] at the interface of CoFeB/MgO. An analysis of the annealing impact on Ta/CoFeB/MgO trilayer composition by means of high-resolution Rutherford backscattering spectroscopy (HRRBS) was discussed in [13], with conclusions that tantalum diffuses into CoFeB, especially for temperatures above 600 K; however, evidence of Ta diffusion into the MgO layer was not observed. The influence of annealing on the magnetic properties of CoFeB/MgO stack was studied for various buffer compositions by Skowronski [14] and Watanabe [15].

Recently, multilayers consisting of a ferromagnet (e.g., CoFeB) layer and a 5d transition metal (e.g., Ta, W, Pt) have attracted significant interest due to their strong spin–orbit coupling. The Spin Hall Effect (SHE) in heavy metals generates a pure spin current, which can excite spin–orbit torque in adjacent ferromagnetic metal or insulator [16,17,18]. It was shown that the magnitude of the spin Hall angle is associated with the crystallographic phase of heavy metals. For instance, the transition between the β and α phases in Ta [19] and in W [20,21] results in a substantial decrease in the spin Hall angle. Similarly, altering the thickness of the Ta seed layer has a significant influence on spin–orbit torques [22,23,24,25]. Precise structural studies and investigations of the interface between Ta and CoFeB facilitate a more nuanced understanding of the dependencies of the spin Hall angle and contribute to the resolution of inconsistencies observed in literature reports.

The present study focuses on the investigation of a CoFeB/MgO stack deposited by means of magnetron sputtering on a Ta buffer layer. The amorphous CoFeB layer enables a smooth interface with the MgO barrier; however, annealing treatment is required to ensure optimal crystallization and enhance the TMR ratio [1]. Although alternative methods of MgO deposition exist, such as atomic layer deposition (ALD) [26], magnetron sputtering is the most widely used in the current state of the art of MTJ’s fabrication. In our previous work [27], the influence of intermixing at the Ta/CoFeB interface on the spin Hall angle was studied, with preliminary θ–2θ X-ray diffraction (XRD) measurements and TEM profiles of the interface, as well as measurements of magnetization and resistivity dependence on temperature. In this study, we present a more detailed structural analysis derived from atomic force microscopy and different diffraction configuration measurements. The present study investigates the microstructure of the Ta seed layers with varying thickness and its influence on interfaces and magnetic properties. The thinnest Ta seed layer exhibits an amorphous structure, while thicker Ta layers tend to crystallize in a low-oriented tetragonal β-Ta phase. CoFeB deposited on the thinnest Ta layer exhibits the largest interface region and the thickest MDL. Temperature-dependent measurements of coercivity field and MDL offer further insights into the morphology of the Ta/CoFeB interface. The MDL temperature measurements are essential for determining the thickness of the active ferromagnetic layer in order to properly calculate the effective field-like and damping-like torques in heavy metal/ferromagnetic systems.

## 2. Materials and Methods

Samples with the following layer structure were prepared on Si/SiO substrate: Ta (*d*)/wedge Co_40_Fe_40_B_20_ (*t*)/MgO 5 nm/Ta 3 nm, with three different Ta buffer thicknesses *d* of 5 nm, 10 nm and 15 nm. The thickness of the CoFeB wedge *t* varied from 0.8 nm to 2.8 nm with a slope of approximately 0.1 nm per 1 cm. The samples were deposited on thermally oxidized Si(001) substrates with an oxide thickness of 100 nm and subsequently annealed at 330 °C for 20 min using the Timaris PVD Cluster Tool system, Singulus, Kahl am Main, Germany. Additionally, samples with only a single Ta layer were prepared. The metallic layers were deposited by DC sputtering with a power of 0.5 kW and an argon flow rate of 300 sccm. Secondary Ion Mass Spectrometry profiles are shown in Figure S1, Supplementary Materials File. The crystallographic microstructure of the layers was investigated by means of X-ray grazing incidence diffraction (XRD GID), rocking curve and θ–2θ scans with different ψ angle between the normal to the sample surface and the diffraction plane. Thicknesses of the layers and interfaces were examined through X-ray reflectivity (XRR) measurements, whereas the morphology and surface roughness were measured with atomic force microscopy (AFM). The magnetic moment, magnetic coercivity and saturation field were determined from vibrating sample magnetometer (VSM) measurements performed in the temperature range of 100–300 K, examples of VSM hysteresis loops shown in Figures S2 and S3, Supplementary Materials File. This paper reports x-ray diffraction and reflectivity studies performed via an X’Pert PRO MPD diffractometer (PANalytical B.V, Eindhoven, The Netherlands). AFM scans were performed via the Ntegra Aura system in SemiContact mode (NTMDT, Apeldoorn, The Netherlands). VSM measurements were performed by a vibrating sample magnetometer LakeShore 7407 (Westerville, OH, USA). Secondary Ion Mass Spectrometry measurements were performed by IonSys 500 ething system (Roth & Rau MicroSystems, Hohenstein-Ernstthal, Germany). Scanning transmission electron microscopy images were taken by FEI Tecnai TF20 X-TWIN (FEG) microscope (Tokyo, Japan).

## 3. Results and Discussion

### 3.1. Microstructure

**Figure 1:**
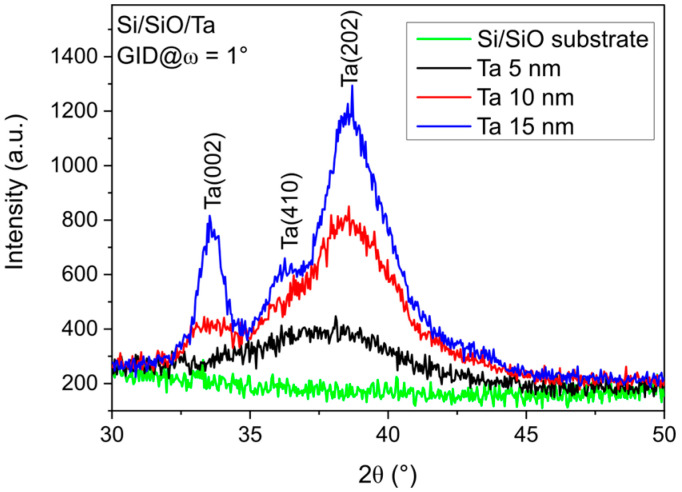
XRD GID profiles of as-deposited single Ta *d* nm layers deposited on Si/SiO_2_ substrates with Ta buffer thicknesses of 5, 10, and 15 nm, measured at an incidence angle of ω = 1°. The Si/SiO_2_ profile is included for comparison.

As illustrated in Figure 1, the XRD GID profiles of as-deposited single Ta layers with Ta thicknesses *d* of 5, 10, and 15 nm are presented. Moreover, the profile for the Si/SiO_2_ substrate is included for comparison. In contrast to the specular θ–2θ profiles reported in Ref. [27], the monocrystalline Si (002) reflection is not visible in the GID profiles due to the measurement geometry, while the polycrystalline Ta-related peaks appear similar. The broad diffraction peaks (002), (410) and (202) of the randomly distributed crystallites correspond to the tetragonal β-phase of tantalum for samples with Ta 10 and 15 nm. The profiles of the 5 nm Ta layer show broad peaks, indicating an amorphous structure.

The resistivity of the Ta buffer layer was measured in Ref. [27] and was found to be approximately 230 µΩ·cm for the 5 nm Ta layer. It decreased to 195 µΩ·cm and 185 µΩ·cm for the thicker 10 and 15 nm Ta layers, respectively. This behavior indicates that the 5 nm Ta layer is amorphous, since resistivity values below 200 µΩ·cm are characteristic of the crystalline β-Ta phase.

**Figure 2:**
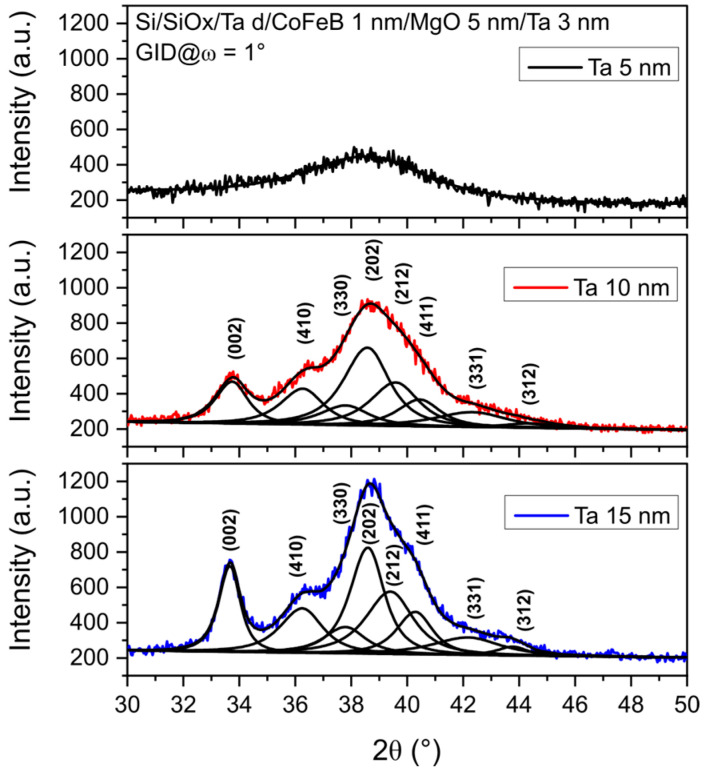
XRD GID profiles and fits of annealed Ta *d* nm/CoFeB 1 nm/MgO 5 nm/Ta 3 nm samples with Ta buffer thicknesses of 5, 10, and 15 nm, measured at an incidence angle of ω = 1°.

A detailed analysis of the θ–2θ profiles of the annealed samples was presented in Ref. [27]. Here, the GID profiles of the whole stack after annealing, which provide a clearer diffraction pattern due to the absence of the MgO (002) peak, are shown in Figure 2.

Fitting of the experimental profiles reveals that the Ta peaks consist of a set of reflections corresponding to the tetragonal β-Ta structure [28]. The relative intensities of the Ta peaks in the θ–2θ [27] and GID profiles are similar, indicating that the Ta seed layer is composed of randomly distributed crystallites. The average grain sizes, calculated from the full width at half maximum (FWHM) of the well-separated Ta (002) peak in the GID pattern, are approximately 9 nm and 6 nm for the 15 nm and 10 nm Ta samples, respectively. Grain sizes calculated from other peaks are smaller, suggesting that Ta growth on the substrate is more favorable along the [002] direction.

Note that the same broad peaks of tetragonal Ta are seen in the profiles of as-deposited samples with only a single Ta layer (Figure 1) and in annealed Ta *d* nm/CoFeB 1 nm/MgO 5 nm/Ta 3 nm structures (Figure 2). The intensities and positions of the Ta peaks remain unchanged after annealing, indicating that Ta layers preserve their primary crystallographic orientations.

**Figure 3:**
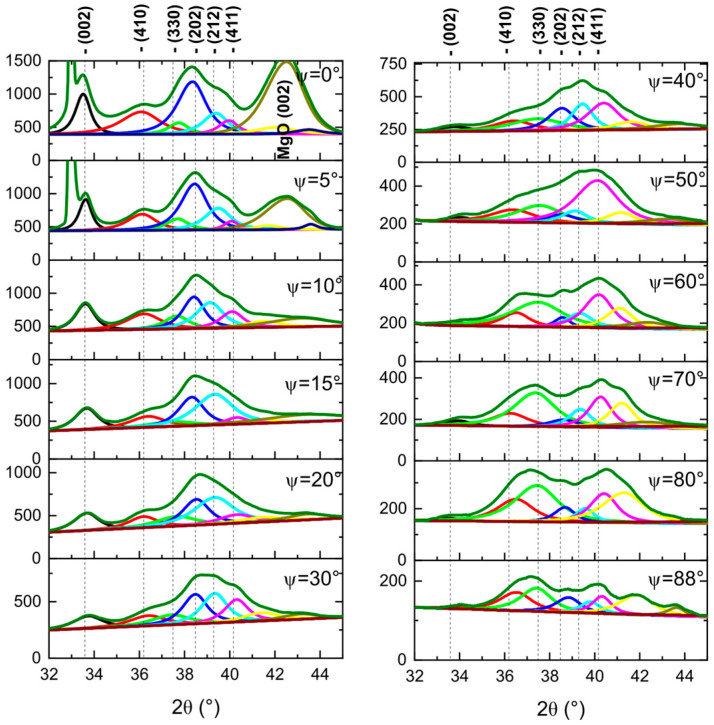
XRD θ–2θ profiles of the annealed Si/SiO/Ta 15 nm/CoFeB 1 nm/MgO 5 nm/Ta 3 nm sample measured at different ψ angles ranging from 0° to 88°.

Additional θ–2θ measurements performed at different ψ angles show relative changes in the intensity of the Ta peaks depending on the sample tilt (see Figure 3).

Compared to the other tantalum peaks, the relative intensity of the Ta (002) peak decreases the most and drops to the background level at ψ angles above 30°. For profiles measured at 65° and 85° ψ, the strongest tantalum peaks are Ta (411) and Ta (330), respectively. These results confirm a weak (002)-oriented texture in the Ta buffer layer, as the interplanar angle between Ta (411) and Ta (002) atomic planes is 65°, and between the Ta (330) and Ta (002) planes it is 90°.

**Figure 4:**
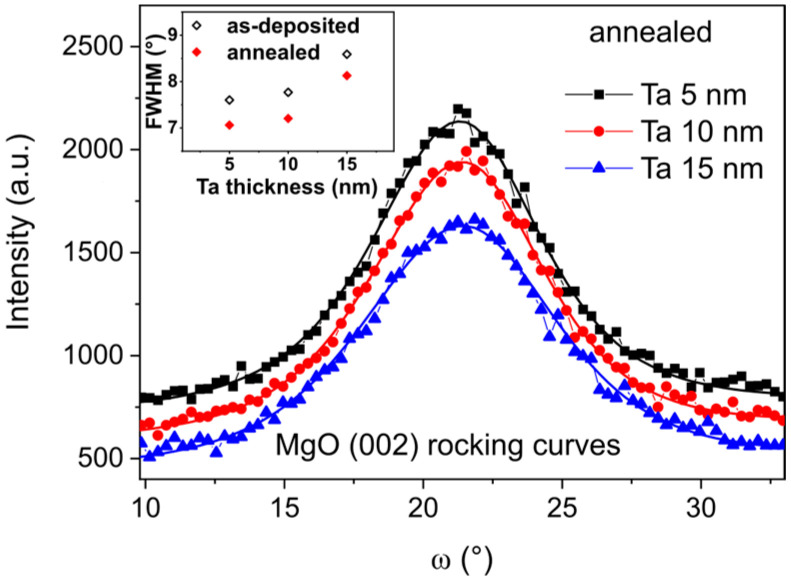
Rocking curve profiles of annealed Ta *d* nm/CoFeB 1 nm/MgO 5 nm/Ta 3 nm samples measured at the MgO (200) reflection. The inset shows the FWHM of the rocking curves for the as-deposited and annealed samples.

**Figure 5:**
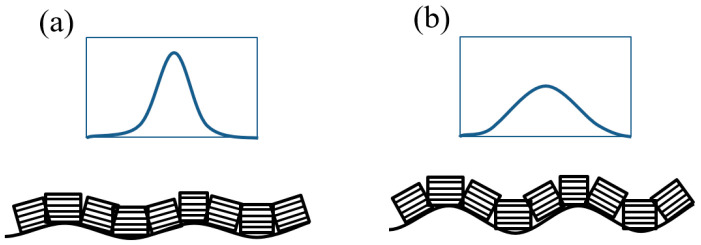
Origin of rocking curve FWHM in relation to surface roughness and crystal grain orientation, smoother surface (**a**), rougher surface (**b**).

To achieve high TMR in magnetic tunnel junctions with a MgO barrier, a highly (001)-oriented texture of fcc MgO is desirable [29]. The presence of a strong MgO (002) reflection in the θ–2θ geometry for ψ = 0 (Figure 3), combined with its suppression in the GID configuration (Figure 2) and for ψ > 10°, indicates a highly (001)-oriented texture of the MgO layer. Rocking curve measurements were performed to evaluate the crystalline quality of the MgO layer. Figure 4 shows the rocking curve profiles as a function of the Ta buffer layer thickness. The lowest FWHM is for 5 nm thick Ta buffer, and the highest FWHM is for 15 nm thick Ta. These results suggest that the (001) texture of MgO is the strongest for the sample with 5 nm Ta buffer and decreases progressively for thicker buffers. This behavior is induced by the morphological roughness of the Ta/CoFeB underlayers, with a rougher underlayer causing greater tilting of the MgO crystallites (Figure 5).

The roughness of the Ta layer affects the orientation of the MgO grains. The morphological roughness of crystallized 15 nm Ta is the highest (see next Section 3.2) and has the strongest influence on the MgO texture. After annealing, as shown in the inset of Figure 4, the FWHM decreases for all samples due to the improvement of MgO texture.

**Figure 6:**
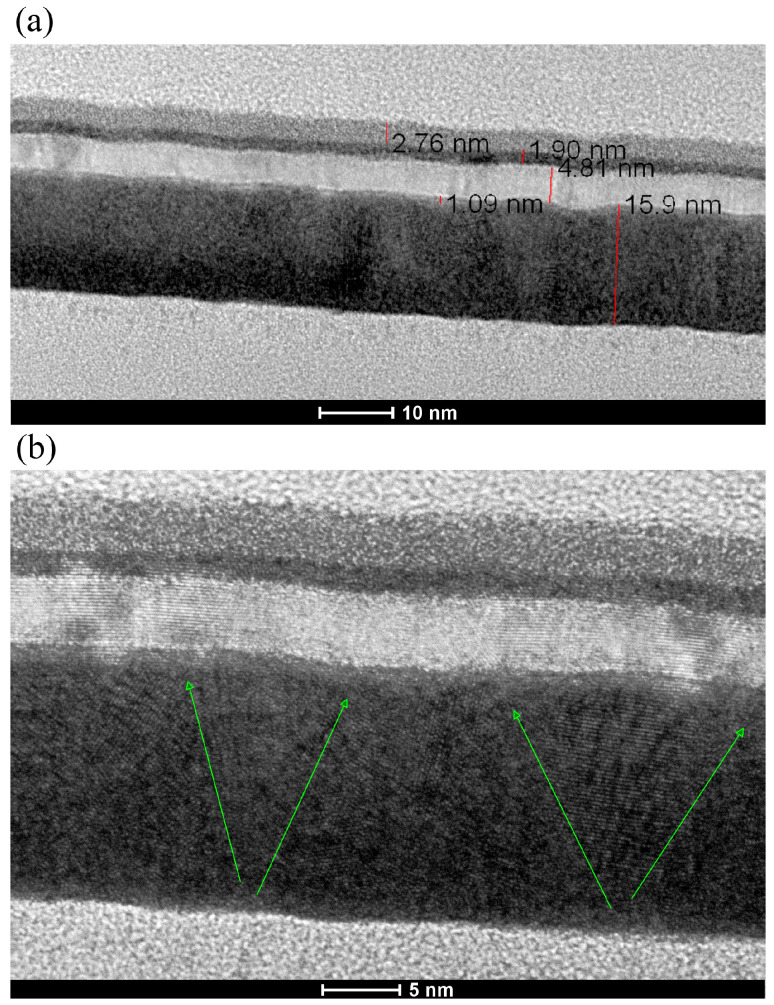
STEM images of the Ta/CoFeB/MgO/Ta/TaO_x_ microstructure, with layer thickness and visible waviness (**a**), growth of conical Ta crystallites (in direction of green arrows) causes topological waviness of the upper layers (**b**).

Figure 6a shows a scanning transmission electron microscopy (STEM) image of the entire stack with 15 nm Ta buffer, along with an example of the thickness of the layers. Another STEM scan shows an example of conical growth of monocrystalline, with a small nucleus on the bottom and increasing size towards the top (Figure 6b), resulting in higher topological roughness, as observed by the AFM.

### 3.2. Surface Roughness and Interfaces

Two measurement techniques were combined to investigate roughness of the surfaces and interfaces: AFM was used to observe the topological roughness, and XRR was used to study interfaces.

**Figure 7:**
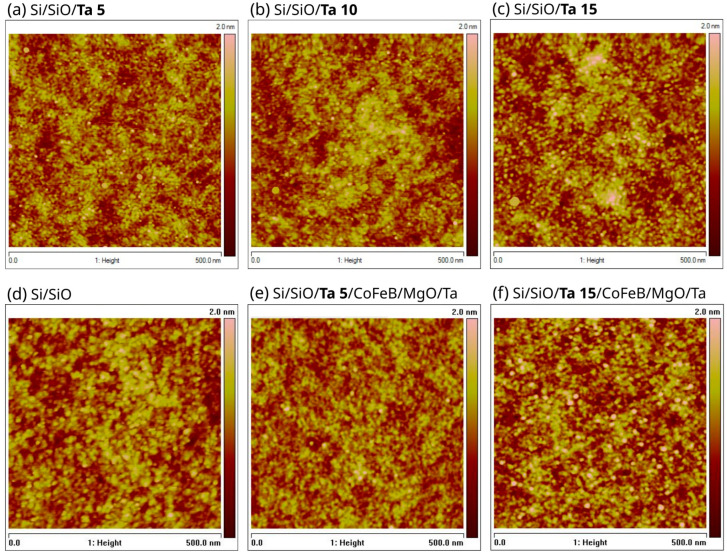
The 500 nm × 500 nm AFM topography images of Ta buffer layers: Ta 5 nm, RMS = 0.23 nm (**a**); Ta 10 nm, RMS = 0.26 nm (**b**); and Ta 15 nm, RMS = 0.29 nm (**c**). AFM images of surfaces directly on SiO_2_, RMS = 0.27 nm (**d**), and on the top of full stacks Ta *d* nm/CoFeB 1.1 nm/MgO 5 nm/Ta 3 nm deposited on Ta buffers with *d* = 5 nm, RMS = 0.24 nm (**e**), and 15 nm, RMS = 0.31 nm (**f**).

Figure 7 presents AFM morphology images scanned directly on the Ta buffers, directly on the wafer seed layer as well as on the top of the entire stack. The Root Mean Square (RMS) roughness for 5 nm buffer Ta was 0.23 nm. The RMS value increases for the 10 nm and 15 nm Ta buffers to 0.26 nm and 0.29 nm, respectively. The roughness measured directly on the Si/SiO_2_ wafer surface was 0.27 nm. Thus, the 5 nm thick amorphous Ta layer decreases the RMS surface roughness, whereas the 15 nm thick Ta layer with larger grains increases this roughness.

**Figure 8:**
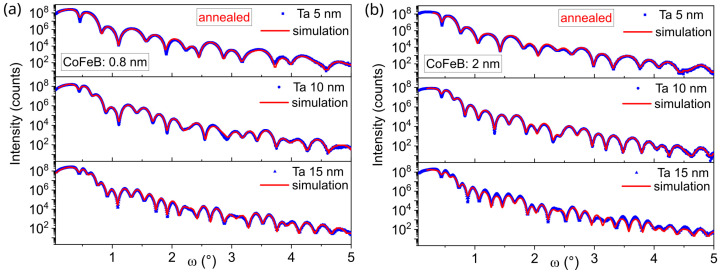
XRR patterns and fits of annealed Ta *d* nm/CoFeB *t* nm/MgO 5 nm/Ta 3 nm samples with Ta buffer layers of 5, 10, and 15 nm for CoFeB thicknesses *t* = 0.8 nm (**a**) and 2.0 nm (**b**).

Figure 8 shows XRR measurements and fits for annealed samples with a nominal CoFeB wedge thickness of approximately 0.85 nm that exhibit perpendicular magnetic anisotropy, and for 2.0 nm samples with in-plane magnetic anisotropy. The thicknesses of the Ta buffer layers obtained from XRR curve fits were 4.9 nm, 9.6 nm and 14.2 nm, which are close to the nominal values. The total Ta/CoFeB interface thickness, consisting of both interdiffusion area and topological roughness (arising from grain sizes and substrate waviness) at the interface for the CoFeB 0.85 nm layer, was 0.55 nm, 0.57 nm, and 0.67 nm, respectively. For the as-deposited samples, roughness at the Ta/CoFeB interface was approximately 0.44 nm. For the 2.0 nm CoFeB layer, roughness decreases for annealed samples to 0.47 nm, and for as-deposited samples, it decreases to 0.35 nm. It is assumed that the roughness at the Ta/CoFeB interface originates from both topological roughness and interdiffusion between the Ta and CoFeB layers; however, specular XRR cannot distinguish between a morphologically rough interface and a compositionally graded one [30].

**Figure 9:**
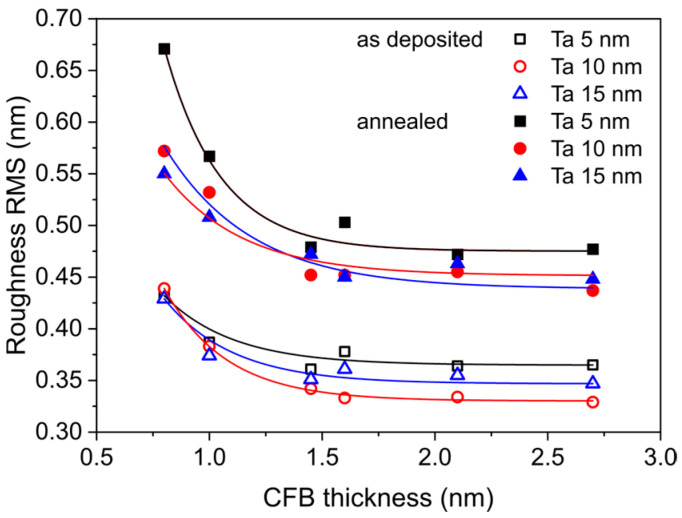
RMS roughness at the Ta/CoFeB interface obtained from XRR fitting for Ta *d* nm/CoFeB *t* nm/MgO 5 nm/Ta 3 nm samples as a function of CoFeB thickness.

Figure 9 shows the roughnesses at the Ta/CoFeB interface as a function of CoFeB thickness *t*. For CoFeB layers thinner than 1 nm, the roughness increases and is highest for annealed sample with a 5 nm Ta seed layer. For thicker CoFeB layers, the roughness decreases, and for CoFeB above 1.5 nm, the roughness is similar for the different Ta buffers. The interface roughness of samples with an amorphous 5 nm Ta buffer is the highest, which confirms high mixing at the Ta/CoFeB interface. The combination of interdiffusion and topological roughness for samples with crystallized Ta layers of 10 nm and 15 nm results in lower interface roughness for the sample with the 10 nm Ta than for the 15 nm Ta sample.

The AFM scans revealed that the thickest (15 nm) Ta buffer exhibited the highest morphological roughness. Reflectivity simulations showed that the Ta/CoFeB interface roughness was highest for the thinnest (5 nm) Ta buffer. The increase in the Ta/CoFeB interface roughness likely occurred through atomic interdiffusion between Ta and CoFeB layers. It is assumed that mixing at the Ta/CoFeB interface is induced by a large negative interfacial enthalpy. The interfacial enthalpy values are −54 kJ/mole of atoms for Fe in Ta and −86 kJ/mole of atoms for Co in Ta [31].

For the as-deposited and annealed samples, the CoFeB/MgO interface roughness obtained from XRR measurements was in the range of 0.20–0.27 nm, without significant changes after annealing. For the thickest (15 nm) Ta buffer, the CoFeB/MgO interface was the widest, which was consistent with the AFM morphological roughness on Ta. The relatively thin CoFeB/MgO interface, compared to the Ta/CoFeB interface, can be explained by the low atomic interdiffusion between these two layers, or even by the repulsion of boron atoms by MgO, as shown by EELS scans in [32]. It stands in contrast to W/CoFeB/MgO stacks, in which a significant boron diffusion into the MgO barrier has been observed. The diffusion of boron into the MgO barrier was also discussed in [33], with the conclusion that boron does not diffuse beyond one or two atomic planes at the CoFeB/MgO interface after annealing at temperatures about 300 °C. Our study shows that the thickness of the CoFeB/MgO interface, as determined by XRR, is comparable to the RMS roughness obtained by AFM measurements. This means that topological roughness is the only source of CoFeB/MgO roughness measured by XRR, in contrast to the Ta/CoFeB interface, where XRR roughness is significantly larger. The topological roughness measured directly on the Ta seed layer transfers through CoFeB to the CoFeB/MgO interface.

### 3.3. Magnetic Properties: Dead Layer and Anisotropy

**Figure 10:**
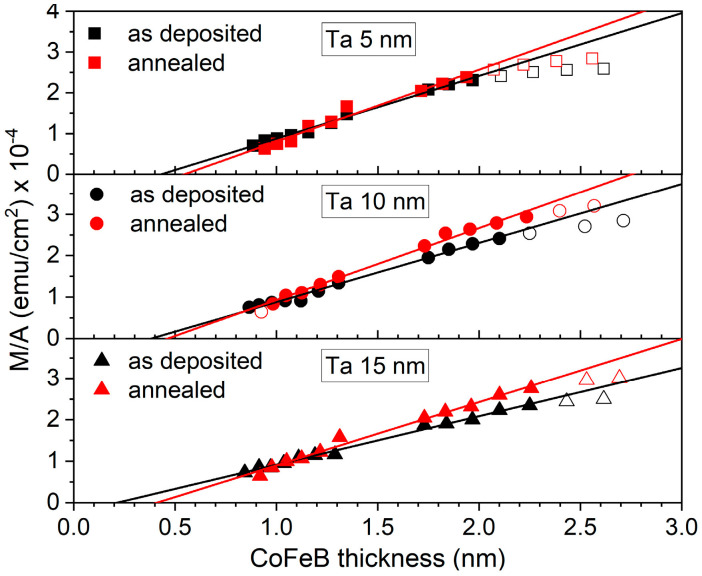
The magnetic moment per unit area of as-deposited and annealed Ta *d* nm/CoFeB *t* nm/MgO 5 nm/Ta 3 nm samples with Ta buffer layers of 5, 10, and 15 nm as a function of CoFeB thickness, with linear approximations. Hollow symbols indicate outliers.

The saturation magnetic moments per unit area versus nominal thickness of the CoFeB layer is presented in Figure 10. From the intersection of the linear fit with the CoFeB thickness axis, the MDL thickness was determined for each buffer [9]. The largest MDL was observed in the sample with the thinnest 5 nm Ta buffer (Table 1). For the as-deposited sample, the MDL reaches 0.43 nm and increases to 0.55 nm after annealing. The MDL decreased with increasing buffer layer thickness, and for the sample with the thickest 15 nm Ta buffer, it reached 0.22 nm and 0.39 nm in the as-deposited and annealed states, respectively. It is worth noting that the largest dead layer was observed for the smoothest Ta buffer, while the thinnest dead layer was found for the roughest Ta buffer. This result can be associated with interfacial mixing, as estimated from XRR at the Ta/CoFeB interface, and the MDL temperature dependence, which will be discussed later.

**Figure 11:**
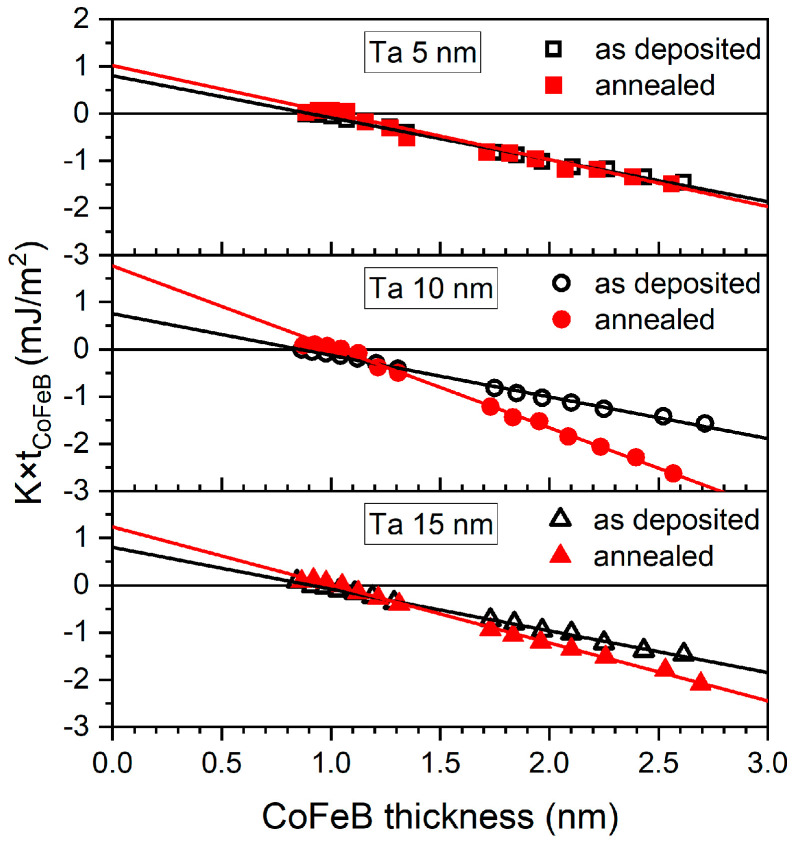
Magnetic anisotropy as a function of CoFeB thickness for as-deposited and annealed Ta *d* nm/CoFeB *t* nm/MgO 5 nm/Ta 3 nm samples with Ta buffer layers of 5, 10, and 15 nm.

Figure 11 shows the dependence of the product of CoFeB layers’ effective anisotropy *K_eff_* and their thickness *t* as a function of CoFeB thickness for as-deposited and annealed samples. The effective anisotropy energy is given by:

*K_eff_* = *K_V_* + 2*K_S_*/*t*, (1)

where *K_V_* is the volume anisotropy and *K_S_* is the surface anisotropy. The nonlinear behavior of *K* × *t* (Figure 11) and M/A (Figure 10) observed at low CoFeB thicknesses (below ~1 nm) can be attributed to strong intermixing between Ta and CoFeB at the interface, which becomes significant when the thickness of the CoFeB magnetic layer is comparable to that of the diffusion zone.

For the as-deposited samples, the volume anisotropy *K_V_* was similar for all three buffers (see Table 1). The interfacial anisotropy was the smallest for the as-deposited samples with a 10 nm Ta layer. For the other samples, *K_S_* remained similar. After annealing, *K_V_* and *K_S_* increased significantly for samples with a thick Ta buffer, due to the CoFeB crystallization. *K_V_* increased over twofold for the sample with a 10 nm Ta layer. Smaller changes were observed for the 15 nm Ta buffer. In the sample with a 5 nm Ta buffer, the anisotropy changes were very small due to the presence of small crystallites and strong intermixing between the CoFeB and Ta, as confirmed by the largest discrepancy between the topological roughness and the XRR interface roughness for the annealed Ta/CoFeB sample with a 5 nm Ta seed layer.

### 3.4. Temperature Dependence of Coercivity and Magnetic Dead Layer

**Figure 12:**
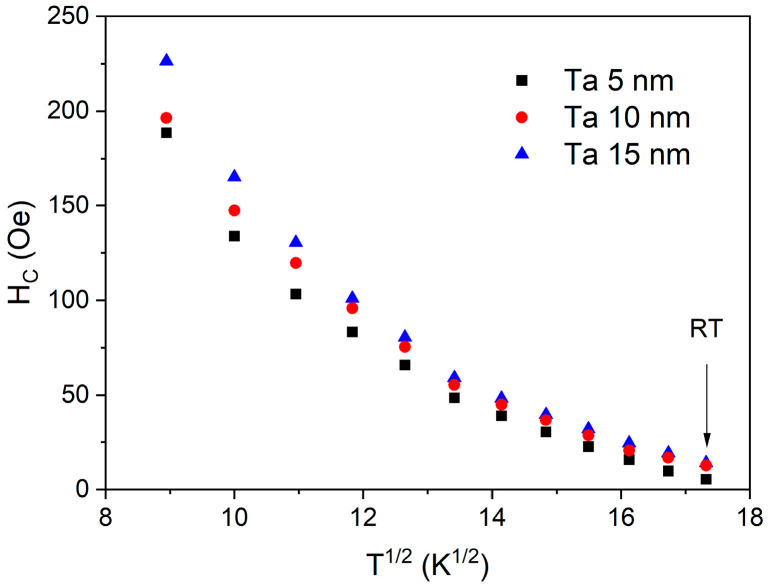
PMA coercivity field for a 0.92 nm CoFeB layer deposited on different Ta buffers, plotted as a function of the square root of temperature.

The temperature dependence of the coercivity field measured for the field perpendicular to the plane is shown in Figure 12. The coercivity field does not follow a linear dependency on the square root of temperature, as can be expected from the Stoner–Wohlfarth model with thermal fluctuation [34]. This model assumes homogenous magnetization, which is not true in the case of thin layers diluted due to interfacial diffusion.

**Figure 13:**
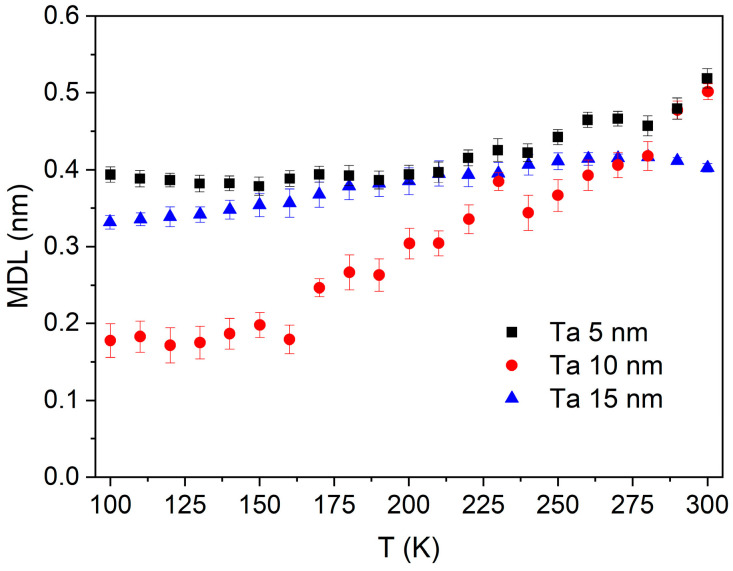
Magnetic dead layer thickness as a function of temperature for annealed Ta *d* nm/CoFeB 1 nm/MgO 5 nm/Ta 3 nm samples with Ta buffer layers of 5, 10, and 15 nm.

The same procedure for determining the MDL, as shown in Figure 10, was repeated for the temperature-dependent VSM measurement, which enabled the extraction of the MDL vs. T, as presented in Figure 13. The smallest changes of MDL with temperature are observed for the sample with a 15 nm Ta buffer. In the sample with 5 nm Ta buffer, the MDL remains almost constant at low temperatures, increasing slightly above around 200 K. Contrary to this, in the sample with a 10 nm Ta buffer, the MDL starts from the lowest value at 100 K, showing the strongest temperature dependence for temperatures above 150 K. This dependence can partially explain why the interfacial effects have the strongest influence on the total spin Hall angle, as determined in Ref. [27] in this temperature range.

**Figure 14:**
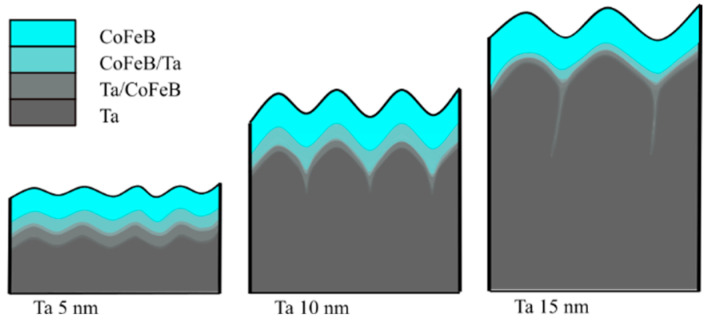
The schematic of diffusion at the Ta/CoFeB interface, where CoFeB/Ta depict paramagnetic volume, becoming ferromagnetic at lower temperatures, and Ta/CoFeB depict lower concentrations of Co and Fe, and hence always paramagnetic.

To help understand the roughness, the influence of annealing, and the MDL behavior of all three samples, we propose a schematic illustration of the diffusion zone at the Ta/CoFeB interface, shown in Figure 14. The largest diffusion zone is observed in the sample with 5 nm Ta buffer, since this Ta seed layer is amorphous (Figure 2), allowing CoFeB to diffuse more easily into Ta at the interface [35]. Crystallization of Ta decreases diffusion at the Ta/CoFeB interface. Diffusion of CoFeB in samples with crystalline Ta primarily occurs at the grain boundaries [36]. In samples with a 10 nm seed layer, Ta tends to crystallize with small grains; thus, there are many shallow CoFeB intrusions into the Ta buffer layer. In the Ta 15 nm sample, the grains in the seed layer are larger; thus, diffusion can proceed mainly along the grain boundaries, with a lower volume ratio than in the sample with a 10 nm seed layer. These assumptions are supported by scanning transmission electron microscopy (STEM) images of multilayer cross-sections presented in [27], where STEM contrast gradually changes along the Ta/CoFeB interface for the 5 nm Ta buffer, while in the case of the 15 nm Ta buffer, the change in STEM contrast is non-linear.

Primarily, CoFeB mixed with Ta at the large grain boundaries becomes magnetic at lower temperatures due to the paramagnetic size effect. The changes are most pronounced in the sample with 10 nm Ta buffer, because Ta crystallization is optimal to reduce the dilution of CoFeB, which exhibits a large paramagnetic MDL at room temperature but becomes almost fully ferromagnetic at low temperatures, including shallow protrusions along grain boundaries. On the other hand, the Ta grains are smaller in the 10 nm buffer than in the 15 nm buffer. As a result, CoFeB does not penetrate the grain boundaries as deeply as in the 15 nm case, although the volume ratio of such intrusions is higher. The overall change of the magnetic dead layer with temperature results from the combined effects observed for 5 nm Ta buffer (change of the paramagnetic region at the interface) and the 15 nm Ta buffer (paramagnetic transition of CoFeB intrusions at grain boundaries).

## 4. Conclusions

We studied the structural and magnetic properties of samples with different thicknesses of the Ta seed-buffer layer. XRD analysis shows that the Ta in the buffers has grown in an amorphous phase or a disoriented tetragonal structure, while the MgO has grown with a highly (001)-oriented texture. The strongest MgO texture was observed for the 5 nm Ta buffer and it decreased successively with the increase in Ta thickness. The smallest roughness was for a 5 nm thick Ta buffer and then increased with the thickness of the tantalum.

At room temperature, the thickest dead layer was observed for the smoothest buffer (5 nm Ta), while for the roughest buffer (15 nm Ta), the dead layer was the thinnest. However, at low temperatures, CoFeB deposited on 10 nm Ta buffer became mostly fully ferromagnetic. Measurements of magnetic dead layer at different temperatures provide valuable insights into interlayer mixing and interface topology, and may also explain the observed temperature anomalies of the interfacial component of the Spin Hall angle.

## Figures and Tables

**Table 1:** Thickness of MDL and CoFeB magnetic properties.

Properties:	As-Deposited			Annealed		
	Ta 5	Ta 10	Ta 15	Ta 5	Ta 10	Ta 15
*t*_MDL_ (nm)	0.43 ± 0.04	0.39 ± 0.05	0.22 ± 0.04	0.55 ± 0.16	0.46 ± 0.05	0.41 ± 0.06
*M*_S_ (emu/cm^3^)	1540 ± 61	1442 ± 44	1180 ± 30	1770 ± 170	1734 ± 60	1510 ± 56
*K*_V_ (J/m^3^) × 10^6^	−0.89	−0.88	−0.88	−0.99	−1.70	−1.22
*K*_S_ (mJ/m^2^)	0.81	0.76	0.80	1.02	1.77	1.24

## Data Availability

The original contributions presented in this study are included in the article/Supplementary Materials. Further inquiries can be directed to the corresponding author.

## Supplementary Materials

The following supporting information can be downloaded at: https://www.mdpi.com/article/10.3390/ma18245558/s1. Figure S1: SIMS profiles of sample deposited on a 5 nm Ta buffer (a) and on a 15 nm Ta buffer (b). Figure S2: VSM hysteresis loops measured along the in-plane easy axis (a), the in-plane hard axis (b) and perpendicular to the sample plane (c). The thickness values are for the CoFeB layer. Figure S3: VSM hysteresis loops measured for a field perpendicular to the sample, at room temperature, for Ta buffer thicknesses of 5 nm (a), 10 nm (b) and 15 nm (c).
