# Supplementary material for: The Ta Seed-Buffer Layer Microstructure and Its Influence on the Magnetic and Structural Parameters of CoFeB/MgO Layers
Source: Materials (Basel). 2025 Dec 11;18(24):5558. doi: 10.3390/ma18245558 (PMC12734422; doi:10.3390/ma18245558)
Supplement: Supplementary file 1 [file materials-18-05558-s001.zip › materials-3914022-supplementary.pdf]

materials

Supplemental material for

The Ta Seed-Buffer Layer Microstructure and Its Influence on the Magnetic and Structural Parameters of CoFeB/MgO Layers

Jarosław Kanak [1], Monika Cecot [1], Witold Skowroński [1], Antoni Żywczak [2,3], Marta Gajewska [2], Jerzy Wrona [4], Wiesław Powroźnik [1] and Maciej Czapkiewicz [1,*]

[1] Institute of Electronics, AGH University of Krakow, Al. Mickiewicza 30, 30-059 Cracow, Poland
[2] Academic Centre for Materials and Nanotechnology, AGH University of Krakow, Al. Mickiewicza 30, 30-059 Cracow, Poland
[3] Department of Mechanical Engineering, Faculty of Engineering, Otemon Gakuin University, Osaka 567-8502, Japan
[4] Singulus Technologies AG, Hanauer Landstrasse 103, 63796 Kahl am Main, Germany
* Correspondence: czapkiew@agh.edu.pl

Secondary Ion Mass Spectrometry

Secondary Ion Mass Spectrometry (SIMS) depth profiles were recorded during ion etching in a Microsystems IonSys 500 tool. SIMS profiles for samples deposited on Ta (5 nm) and Ta (15 nm) are shown in Figure S1 (a and b), respectively. The expected element sequence of the layers can be identified - the top Ta layer, Mg, Co and Fe, and the bottom Ta buffer layer. Although the overall ion counts are relatively low, the obtained data, together with our previous measurements, consistently indicate that the interface with the amorphous Ta buffer is more diffuse than that of the samples deposited on 15 nm thick Ta, while MgO/CoFeB interface is sharp in both cases.

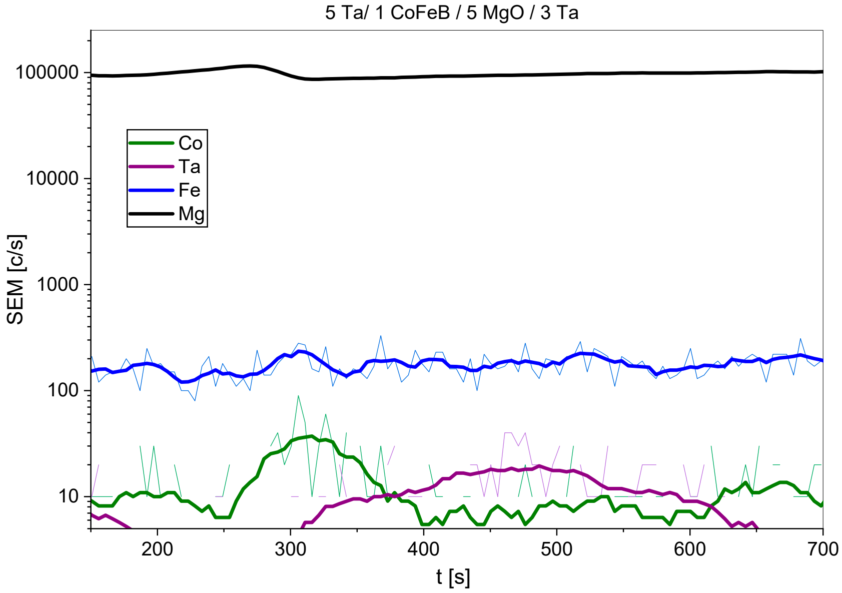

(a)

 https://doi.org/10.3390/ma18245558

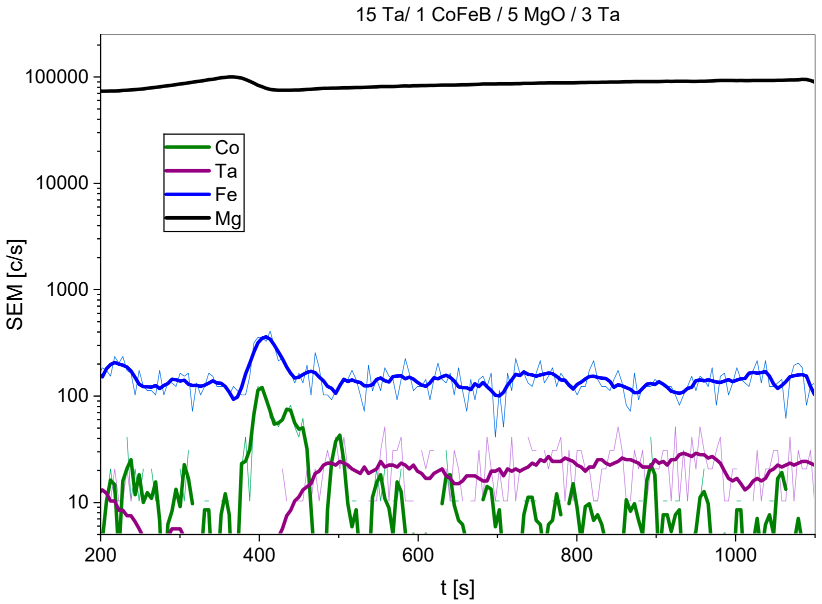

(b)

Figure S1. SIMS profiles of sample deposited on a 5 nm Ta buffer (a) and on a 15 nm Ta buffer (b).

Magnetization hysteresis loops

The magnetic properties of the CoFeB layer were examined using vibrating sample magnetometry (VSM) measurements of rectangular stripes, with a magnetic field oriented in plane or perpendicular to the plane. Thick CoFeB layers (t > 1 nm) are characterized by an in-plane anisotropy, with an easy axis along the longer edge. Saturation magnetization and anisotropy energy were determined from measurements taken along hard axis (along the shorter edge of the stripe for thin CoFeB layers, or perpendicular to the plane, for thick ones). An example of magnetic moment measurements for annealed sample with 5 nm Ta buffer are shown in Figure S2. Figure S3 presents a comparison of PMA hysteresis loops for annealed samples deposited on 5 nm, 10 nm and 15 nm Ta buffer, with three different CoFeB thicknesses.

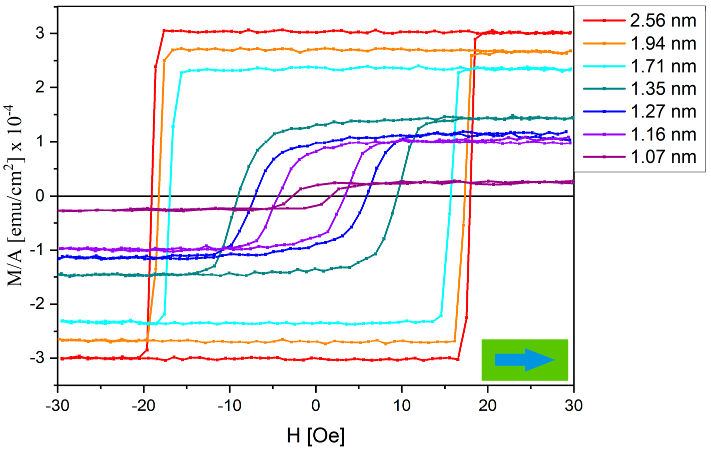

(a)

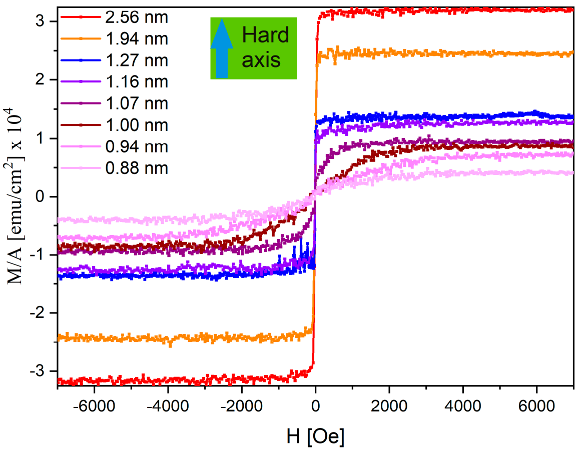

(b)

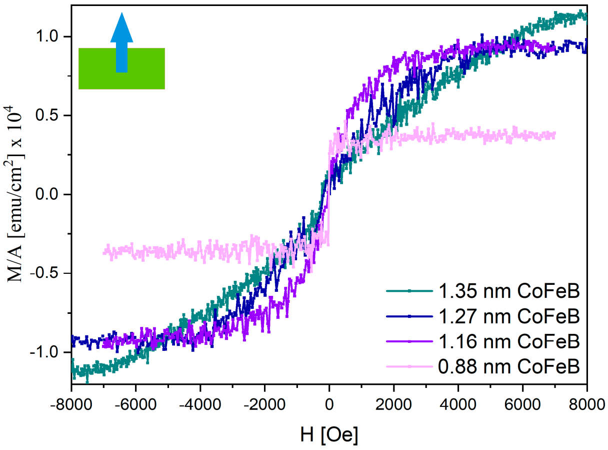

(c)

Figure S2. VSM hysteresis loops measured along the in-plane easy axis (a), the in-plane hard axis (b) and perpendicular to the sample plane (c). The thickness values are for the CoFeB layer.

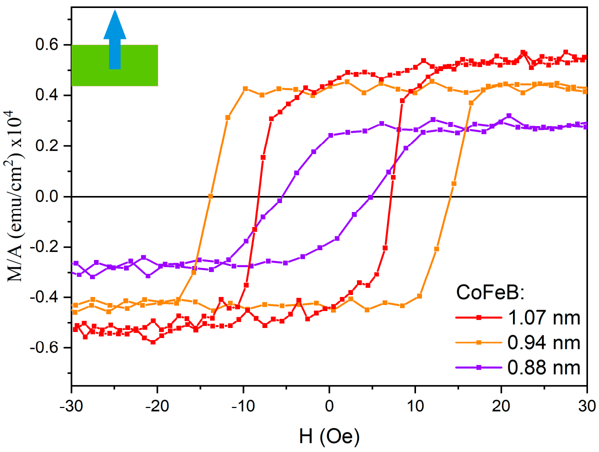

(a)

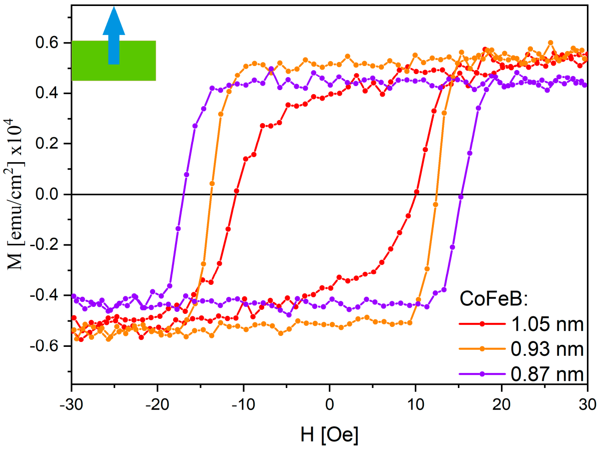

(b)

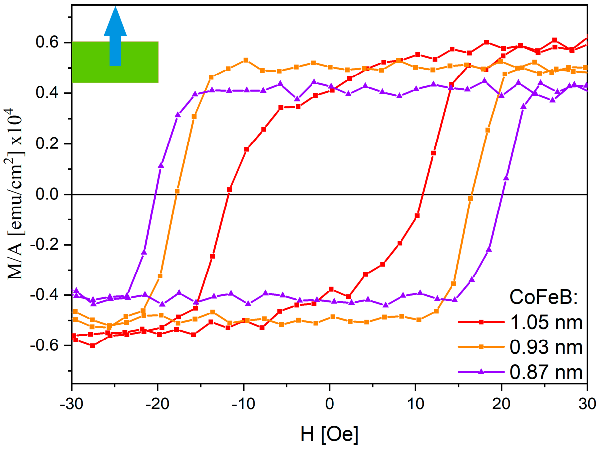

(c)

Figure S3. VSM hysteresis loops measured for a field perpendicular to the sample, at room temperature, for Ta buffer thicknesses of 5 nm (a), 10 nm (b) and 15 nm (c).
